# A Graded Multifunctional Hybrid Scaffold with Superparamagnetic Ability for Periodontal Regeneration

**DOI:** 10.3390/ijms19113604

**Published:** 2018-11-15

**Authors:** Simone Sprio, Elisabetta Campodoni, Monica Sandri, Lorenzo Preti, Tobias Keppler, Frank A. Müller, Nicola M. Pugno, Anna Tampieri

**Affiliations:** 1 Institute of Science and Technology for Ceramics-National Research Council (ISTEC-CNR), Via Granarolo 64, 48018 Faenza, Italy; elisabetta.campodoni@istec.cnr.it (E.C.); lorenzo.preti@istec.cnr.it (L.P.); 2 Laboratory of Bio-Inspired & Graphene Nanomechanics, Department of Civil, Environmental and Mechanical Engineering, University of Trento, Via Mesiano 77, 38123 Trento, Italy; nicola.pugno@unitn.it; 3 Otto Schott Institute of Materials Research, Friedrich Schiller University, Löbdergraben 32, 07743 Jena, Germany; tobias_keppler@yahoo.de (T.K.); frank.mueller@uni-jena.de (F.A.M.); 4 School of Engineering and Materials Science, Queen Mary University of London, Mile End Road, London E1 4NS, UK; 5 Ket-Lab, Edoardo Amaldi Foundation, Italian Space Agency, Via del Politecnico, 00133 Rome, Italy

**Keywords:** biomimetic hybrid scaffold, biomineralization, electrospinning, periodontal regeneration, collagen, superparamagnetic hydroxyapatite

## Abstract

The regeneration of dental tissues is a still an unmet clinical need; in fact, no therapies have been completely successful in regenerating dental tissue complexes such as periodontium, which is also due to the lack of scaffolds that are able to guide and direct cell fate towards the reconstruction of different mineralized and non-mineralized dental tissues. In this respect, the present work develops a novel multifunctional hybrid scaffold recapitulating the different features of alveolar bone, periodontal ligament, and cementum by integrating the biomineralization process, and tape casting and electrospinning techniques. The scaffold is endowed with a superparamagnetic ability, thanks to the use of a biocompatible, bioactive superparamagnetic apatite phase, as a mineral component that is able to promote osteogenesis and to be activated by remote magnetic signals. The periodontal scaffold was obtained by engineering three different layers, recapitulating the relevant compositional and microstructural features of the target tissues, into a monolithic multifunctional graded device. Physico-chemical, morphological, and ultrastructural analyses, in association with preliminary in vitro investigations carried out with mesenchymal stem cells, confirm that the final scaffold exhibits a good mimicry of the periodontal tissue complex, with excellent cytocompatibility and cell viability, making it very promising for regenerative applications in dentistry.

## 1. Introduction

The tooth is a complex organ made of highly mineralized tissues (i.e., alveolar bone, cementum, dentin, enamel) where the non-mineralized periodontal ligament (PDL) connects the alveolar bone to the cementum, and ensures tooth functionality and stability. Tooth diseases are extremely widespread worldwide, particularly periodontitis, which is the second most common chronic disease, with a prevalence that is similar to cardiovascular disease and diabetes [1,2]. Severe periodontitis is characterized by tissue resorption involving both the alveolar bone and cementum, which provokes the loss of the tooth. In these cases, spontaneous regeneration does not usually occur on a clinically predictable basis, and thus the current approaches for the treatment of periodontal diseases are mostly based on preservative and conservative approaches. However, over the last few decades, ever-increasing attention has been focused on dental regenerative and reconstructive therapies, rather than resective surgery. Among various approaches, guided periodontal regeneration has been the only successful therapeutic approach to date [3,4]. Guided tissue regeneration focuses on the use of bioactive and bio-resorbable scaffolds acting as 3D porous matrices, promoting cell attachment, proliferation, and specific differentiation, thus sustaining the formation of new healthy tissues with appropriate texture and biomechanical competence.

As in bone, the phenomena active in the formation of the different dental tissues involve collagen and proteoglycans as an assembling macromolecular matrix, mineralized with hydroxyapatite nano-crystals (HA), and organized into complex fibrous constructs with graded morphologies and structures. Alveolar bone surrounds the roots of teeth, to provide support, creating what is commonly called a “socket” where the PDL connects the alveolar bone to the cementum. Cementum is an avascular tissue, whose mineralization degree is about 50%, organized into two distinct parts: acellular (or primary) and cellular (or secondary) cementum. Cellular cementum forms bundles around the fibres of the PDL, thus entrapping cementoblasts, which are similar to osteocytes in bone. It has a complex architecture described as a twisted plywood structure with an alternating lamellar pattern, so that it may be represented as a woven fabric with wide radial and narrower circumferential collagen fibres. However, the implantation of different bone grafts has not always been successful for the re-establishment of the periodontal attachment apparatus adequately, due to undesired cellularization, and due to ineffective organization of the collagen fibers in the new cementum obtained, which does not permit firm anchoring to the root surfaces [5].

To date, approaches to periodontal regeneration are still in their infancy, and the main issue is the difficulty in driving the correct development of the new dental tissues. In fact, the events associated with periodontal regeneration are extraordinarily complex, and they require the participation of all cellular components of the periodontium: fibroblasts for soft connective tissues such as PDL, cementoblasts for cementogenesis, osteoblasts for bone, and endothelial cells for angiogenesis [6,7]. All of these cell lineages must correctly interact with each other, as well as with a variety of molecules of the extracellular matrix (ECM). Therefore, to effectively drive the correct metabolic processes, periodontal scaffolds should act as instructors for stem cells, possibly re-creating biomimetic chemical and morphological environments at the healing site.

In previous studies, nanostructured polymer membranes based on polymethyl methacrylate were proposed for periodontal regeneration. These structures can be modified adding foreign ions such as zinc and calcium, to promote osteoconductivity and osteogenic ability, thanks to the precipitation of HA biomimetic nanocrystals [8]. Other strategies that were evaluated to treat periodontitis were the use of diode lasers or the use of desiccant agents as an adjunct to scaling and root planing. A diode laser therapy combined with scaling and root planing revealed an improvement in clinical, microbial, and inflammatory parameters after a year of treatment of patients with generalized aggressive periodontitis, highlighting higher levels of clinical effects compared to scaling and root planing alone [9]. Furthermore, the same results have been found by Isola et al. using a chemotherapeutic agent, a desiccant agent in combination with scaling and root planing in the treatment of chronic periodontitis [10].

Scaffolds with 3D architecture that are suitable for cell colonization and differentiation were obtained by several techniques such as electrospinning and freeze-drying techniques. Electrospinning is a versatile technology used to produce highly porous and thin nonwoven mats of nanometric fibrils, also by using bio-erodible polymers embedding inorganic particles [11,12]. Freeze-drying of hydrogels is a powerful technique that can be directed to obtain 3D scaffolds with highly interconnected porous morphology, by controlling the sublimation kinetics of the solvents [13,14]. In attempting to generate hybrid, mineralized gels, in a previous study, a bio-inspired process was applied to obtain the heterogeneous nucleation of nanocrystalline ion-doped apatite onto self-assembling Type I collagen fibrils (Coll) [15,16,17]. The process resulted in scaffolds with graded mineralization and morphology, mimicking the osteochondral tissue complex comprising bone, tidemark, and articular cartilage. The nucleation of the mineral phase was obtained thanks to the presence of free carboxylic groups exposed by the organic macromolecular matrix as sites for the linking of Ca^2+^ ions, and in turn, the formation of mineral nuclei featuring very low crystallinity and lattice substitutions with ions-enhancing bioactivity, such as Mg^2+^, CO_3_^2−^, and Sr^2+^, under the constraints imposed by various control mechanisms exerted by the template at the multi-scale level [17]. The porous hybrid scaffolds obtained by such methods were evaluated in various pre-clinical and clinical studies, and their close mimicry with host tissues was a key factor for excellent regenerative abilities [18,19,20]. The composition of various mineralized tissues with bio-relevant ionic substitutions can be reproduced by wet neutralization synthesis methods, enabling the control of the amount of ions occupying calcium or phosphate sites [21,22,23]. In previous studies, both ferrous and ferric ions were introduced in controlled ratios with calcium, thus generating a particular configuration of Fe^2+^ and Fe^3+^ ions in the apatite structure whose interactions induce superparamagnetic properties into the mineral phase [24,25]. This phase could be also obtained as a superparamagnetic inorganic phase in hybrids obtained by bio-inspired mineralization, similar to that described above [26]. The use of hybrid magnetic biomaterials is today considered to be a very promising approach for boosting tissue regeneration, either by direct magnetic stimulation of the osteogenesis process, or by the on-demand activation of functionalized magnetic nanoparticles carrying drugs or other relevant bioactive molecules [26,27]. In fact, besides the direct effect of applied magnetic fields, the substitution of calcium with iron ions was previously shown to enhance the bioactivity of apatites and hybrids, in terms of cell proliferation and osteogenic ability [27].

Intensive work has been devoted to the fabrication of bone and osteochondral scaffolds for orthopedic applications; particularly, biomimetic devices have demonstrated great effectiveness in regenerating multifunctional tissues. Nevertheless, few attempts have been made so far to develop scaffolds that are intended for the regeneration of dental tissues, and in particular, the periodontium. Hence, in this work, we aim to develop a biomimetic periodontal scaffold, recapitulating the different compositional and structural features of mineralized and non-mineralized tissues composing the periodontium. Furthermore, we aim to validate a novel approach of generating scaffolds with superparamagnetic properties that can be activated by remote signaling on demand, to push tissue regeneration; this can be particularly useful in dental applications where reduced tissue porosity and the possible occurrence of infections can jeopardize or retard the regenerative process. 

To this purpose, different fabrication techniques were adopted to develop three different constructs mimicking the alveolar bone, PDL, and the cementum tissues; the constructs are then engineered into a final monolithic hybrid body presenting a graded mineralization extent and textured fibrous structures. In particular, alveolar bone is obtained through a biomineralization process in which self-assembly and organization of Type I collagen (Coll) is carried out in the presence of Ca^2+^, Fe^2+^/Fe^3+^, and PO_4_^3−^ ions to achieve the heterogeneous nucleation of iron-doped hydroxyapatite (FeHA) in amounts mimicking the mineral content of alveolar bone (i.e., ~70 wt %). Cementum is developed through the electrospinning of a bio-erodible polymer mineralized with FeHA to produce very thin and mineralized layers of fibers closely reproducing the structure of the natural cementum tissue. The non-mineralized PDL is obtained by pH-driving the self-assembling and supramolecular organization of Type I collagen nano-fibrils, and then subjected to a cross-linking reaction and freeze-drying process, to obtain a mechanically competent fibrous scaffold.

Finally, assembly of the three layers into the final periodontal scaffold is made and optimized to achieve a good level of interpenetration, preventing delamination and preserving the tissue-mimicking morphology in all of the layers. The obtained constructs are characterized in terms of physico-chemical and morphological properties, and subjected to preliminary in vitro cell tests to assess their biocompatibility. 

## 2. Results

### 2.1. Hybrid Scaffold Mimicking the Alveolar Bone 

The scaffold mimicking the alveolar bone is obtained as a FeHA/Coll hybrid, with a mineral/organic phase weight ratio of 70:30, reproducing the mineralization extent and porous structure of bone tissue. The partial substitution of calcium ions with iron ions (20% mol Fe/Ca) obtained at 40 °C gives the mineral phase superparamagnetic ability; genipin was introduced at a genipin/Coll ratio of 2 wt % as a cross-linking agent to increase the stability of the scaffold in aqueous media. Finally, the freeze-drying process is effective in the organization of the composite fibers into a 3D network with cell-conducive porosity.

**Figure 1:**
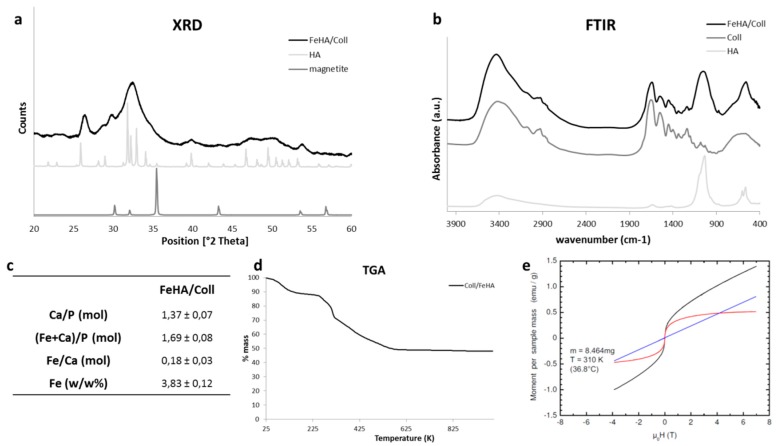
(**a**) XRD spectra of the FeHA/Coll scaffold compared to HA and magnetite powders; (**b**) FTIR analysis of the FeHA/Coll scaffold compared to collagen and HA powder; (**c**) ICP features of FeHA/Coll; (**d**) Thermal decomposition profile (TG) of the FeHA/Coll scaffold; (**e**) Magnetization curves at *T* = 310 K of FeHA/Coll scaffold measured curves in black, nonsaturating and saturating paramagnetic contributions, in blue and red respectively.

The mineral phase nucleated on the assembling collagen fibrils is hydroxyapatite, according to the Powder Diffraction File (PDF) card #09-0432 (see Figure 1a), with no detectable traces of iron oxides, which are recognized as cytotoxic, particularly superparamagnetic Fe_3_O_4_ or Fe_2_O_3_ (magnetite or maghemite). The broad peaks evidenced in the X-ray diffraction pattern (XRD) reported to a mineral phase with very small crystal domains, as an effect of the low synthesis temperature, and the possible incorporation of foreign ions in the apatite structure, provoking structural destabilization. Noticeably, the 002 reflection was particularly enhanced, showing a preferential crystal development along the *c* axis of the hexagonal lattice of hydroxyapatite. Fourier-Transform InfraRed spectra (FTIR) in Figure 1b show typical absorption bands related to the PO_4_ group in the apatite structure (e.g., at 1032, 565, 601 cm^−1^); the very low splitting factor obtained by the FTIR spectra further confirmed the disordered crystal state of the mineral phase. Band resolution relative to HA is low, and the typical behavior of non-stoichiometric and low crystalline HA is developed by a biomineralization process at low temperature. The analysis of chemical composition carried out by Inductively Coupled Plasma-Optical Emission Spectrometry (ICP-OES) is reported in Figure 1c, highlighting a reduced calcium content, with respect to stoichiometric HA (i.e., ~1.67), and a slightly higher (Ca + Fe)/P ratio. The reduced calcium content confirms the occurred iron substitution in the apatite lattice, while, on the other side, the higher cation/anion ratio suggests that some of the iron ions were incorporated into the non-ordered crystal environment, typical of low-crystallinity apatites [25]. The lesser amount of Fe detected in the hybrid composite, in comparison with the starting nominal concentration, can be ascribed to the competition between Fe and Ca ions to occupy the same crystal site; however, a synthesis temperature of 40 °C was effective to favor such substitution and to obtain hybrids with superparamagnetic abilities. 

The weight loss detected by Thermogravimetric Analysis (TGA) suggests that the amount of the mineral phase nucleated on collagen fibers was ~60 wt % (Figure 1d), a value that was close to the mineralization extent of natural bone. 

The magnetization curves (see Figure 1e) at *T* = 310 K (black curves) can be decomposed into two components: (i) the background (blue lines), increasing linearly with the field, which is typical for paramagnetic materials far away from saturation, can probably be assigned to Coll; and (ii) the red curves following a Brillouin function describing the superparamagnetic behavior of FeHA. The low magnetization value (0.5 emu/g) can be ascribed to the highly disordered structure of the mineral phase FeHA, as imposed by the Coll template, and the consequent poor coordination level of the iron ions in its structure. FeHA/Coll showed almost no hysteresis at zero field, which is an indicator for the absence of ferromagnetism.

**Figure 2:**
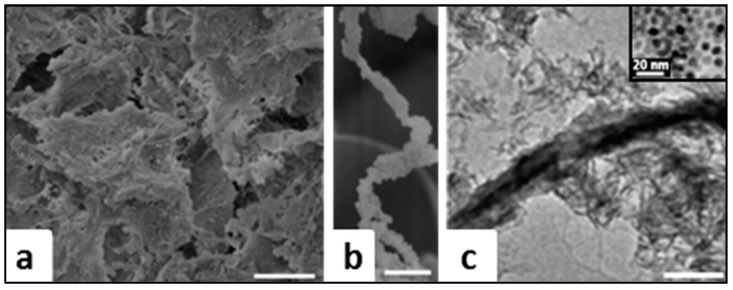
(**a**,**b**) SEM images of FeHA/Coll scaffold at different magnification (**c**) TEM images of FeHA/Coll scaffold compared to magnetite TEM image (inset). Scale bars: (**a**) 100 um, (**b**) 5 um, (**c**) 200 nm.

Microscopic images of FeHA/Coll scaffold obtained by Scanning Electron Microscopy (SEM) are reported in Figure 2a, showing a 3D fibrous architecture with a network of micro-macro pores and very small mineral nuclei linked to the Coll fibers. The rough surface can be ascribed to the presence of the mineral phase also inside the Coll fibers, due to the mineralization process occurring at the same time as the supramolecular assembling of the original collagen nano-fibrils into thicker fibers [15] (Figure 2b). A deeper insight of the mineral nanophase is given by Transmission Electron Microscopy (TEM) analysis (Figure 2c), showing the preferential orientation of FeHA nanocrystals along their *c*-axis, in agreement with the XRD analysis. Extremely low crystallinity, extent, and orientation of the mineral phase were all features confirming that the nucleation of the mineral phase occurred under the physical constraints imposed by the collagen matrix, thus giving a hybrid construct that closely mimicked the composition and multi-scale structure of the so-called woven bone [28]. High-resolution TEM images confirmed the absence of any particles with significant different morphology, which could be ascribed to secondary nanophases such as iron oxides (see inset) and that confirmed the phase purity of the FeHA/Coll hybrid. 

### 2.2. Collagenous Scaffold Mimicking the Periodontal Ligament

**Figure 3:**
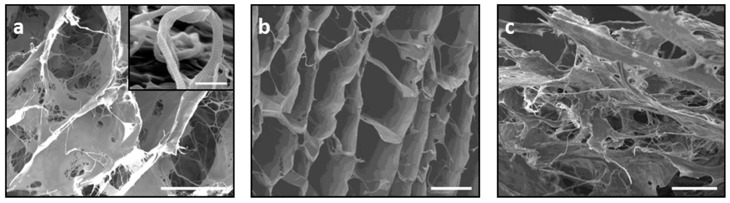
SEM images of Coll scaffold at (**a**) high magnification; (**b**) medium magnification; (**c**) low magnification. Scale bars of (**a**–**c**) is 100 µm. In the inset of Figure 3a is showed a SEM image of Coll fibres at high magnification. Scale bar of inset is 500 nm.

To produce the thin layer mimicking the periodontal ligament, the acid Coll suspension was neutralized with 0.5 M NaOH, bringing the pH up from 3.5 to 5.5, which corresponds to the isoelectric point of collagen, enhancing the supramolecular assembling of the collagen fibrils. The so-obtained Coll gel was then cross-linked with 2 wt % BDDGE (1,4-butanediol diglycidyl ether) and thin membranes were developed by a tape-casting technique matched with an air-drying process under environmental conditions. The scaffold morphology was observed by SEM, showing a homogeneous structure condensed in sheets, as well as the single fibers with typical alternating light and dark bands corresponding to the 40-nm gaps between pair of aligned collagen triple helices (see inset), typical of collagen fibers (Figure 3a). Comparing the collagen structure in the presence or not of cross-linking, the influence of cross-linking was also clearly seen in the morphology. In the absence of cross-linking (Figure 3b), the collagen scaffold showed an orderly and regular 3D morphology, due to the high mobility of the fibers that could be displaced by the growth of ice crystals during freeze-drying. In this way, the pore structure is built up from parallel collagen layers connected by collagen walls. Conversely, the cross-linking reaction reduced the polymer mobility during the freeze-drying process, so that the 3D geometry was less influenced by the ice growing during the freeze-drying, and more by the component of the molecules packing resulting from the cross-linking process (Figure 3c). Thus, the cross-linked fibers were too stiff to be displaced by the growth of ice crystals, and the final dried structure showed more sheet-like crystals with wide but few pores, better mimicking the natural PDL.

### 2.3. Hybrid Scaffold Mimicking the Cementum

For the development of the cementum part, FeHA nanoparticles synthesized at 40 °C following a process previously reported [21], were conjugated with cellulose acetate (CA) to obtain FeHA/CA cementum-like scaffolds by an electrospinning process.

**Figure 4:**
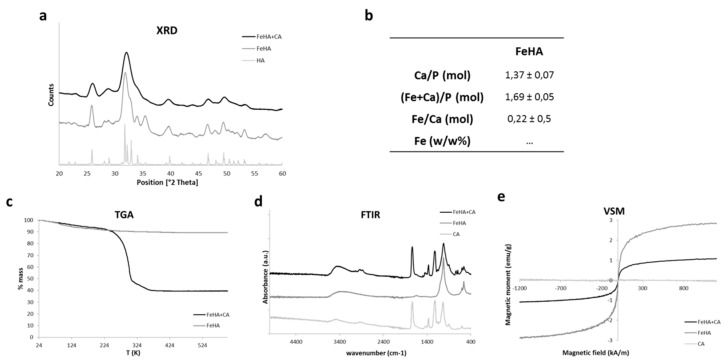
(**a**) XRD spectra of the FeHA/CA scaffold compared to FeHA and HA powders; (**b**) ICP features of FeHA; (**c**) Thermal decomposition profile (TG) of the FeHA/CA scaffold compared to FeHA powder; (**d**) FTIR analysis of the FeHA/CA scaffold compared to the FeHA powder and cellulose acetate; (**e**) Superparamagnetic contribution of the FeHA/CA scaffold compared to FeHA powder.

The XRD patterns of FeHA and FeHA/CA are shown in Figure 4a and they revealed a low-crystalline apatite phase with a peak broadening related to the constraints of crystal development imposed by the collagen matrix during the biomineralization process, in comparison with stoichiometric HA synthesized by a wet process at the same temperature. Even if in smaller amounts, in FeHA, the presence of magnetite was notable, with its main peak at 2θ = 35.4° and 57.3°; its presence was due to the temperature increasing, which caused not only the increase of apatite crystallization, but also the formation of magnetite. The addition of non-crystalline CA made it difficult to detect the entire XRD pattern of FeHA showing a lower-crystalline phase, but maintaining the structure of the apatitic phase and the XRD main profile (Figure 4a).

The ICP analysis, carried out on FeHA nanoparticles (Figure 4b), revealed a Ca/P molar ratio that was lower than the one of stoichiometric HA, but the (Ca + Fe)/P ratio was very close to the theoretical value of 1.67, confirming the replacement of calcium with iron. The slightly higher ratio is due to the incorporation of iron ions in non-ordered positions that could generate a small amount of magnetite in the mineral phase. Furthermore, the yield of iron ion doping was effective, as almost all iron nominally introduced as a reagent was found in the HA lattice (precisely 90%). The TGA analysis reported in Figure 4c showed the degradation of the organic component, particularly a weight loss with a maximum rate at 314 °C addressing the degradation of the CA chains, following a slight initial weight loss due to the evaporation of residual absorbed water. Finally, the total inorganic residue corresponding to the mineral component of the scaffold was ~50%, which was very close to the designed mineralization extent, and close to the level of biological cementum. The FTIR spectra of FeHA/CA in Figure 4d confirm the successful incorporation of FeHA nanoparticles into the electrospun CA structure, attested by the absorption bands that are typically ascribed to apatite (i.e., at ~1032 cm^−1^, 604 cm^−1^, 562 cm^−1^) and to CA (i.e., 3472 cm^−1^ attributed to O-H stretching, 2954 and 2891 cm^−1^ attributed to C–H stretching in CH_3_ and CH_2_ groups), 1754 and 1651 cm^−1^ (symmetric and asymmetric stretching of C=O), 1441 cm^−1^ (bending of CH_2_), and 1375 cm^−1^ (bending of CH).

The magnetization curves and the results observed for FeHA, CA, and FeHA/CA are shown in Figure 4e. The curves highlighted no magnetic properties of the organic component, and the typical superparamagnetic behavior for FeHA and its composite, because the amplitude of hysteresis at zero-field was around zero. The synthesis temperature of 40 °C induced FeHA crystal organization, giving a high magnetization value (2.85 emu/g), and the incorporation of the mineral phase with CA through the electrospinning technique did not interfere with its magnetic properties, because also the composite revealed a suitable value of magnetization (1.09 emu/g) considering the reduced mineral amount (i.e., 50%). The magnetization value was higher than FeHA/Coll, confirming again the different role of collagen and cellulose acetate in the biomineralizion process; in fact, the superparamagnetic properties of the obtained composites, as related to the crystal organization and ordering, were affected by the structural confinement exerted by the protein template on the mineral phase, preventing free crystallization and growth, but reducing the structural order at the lattice level of the iron species. This condition is not present in FeHA/CA, because the two different components were simply mixed and not integrated by a heterogeneous nucleation process, such as in biomineralization; thus the reduction of the magnetization was only due to the reduced amount of mineral phase in the composite.

**Figure 5:**
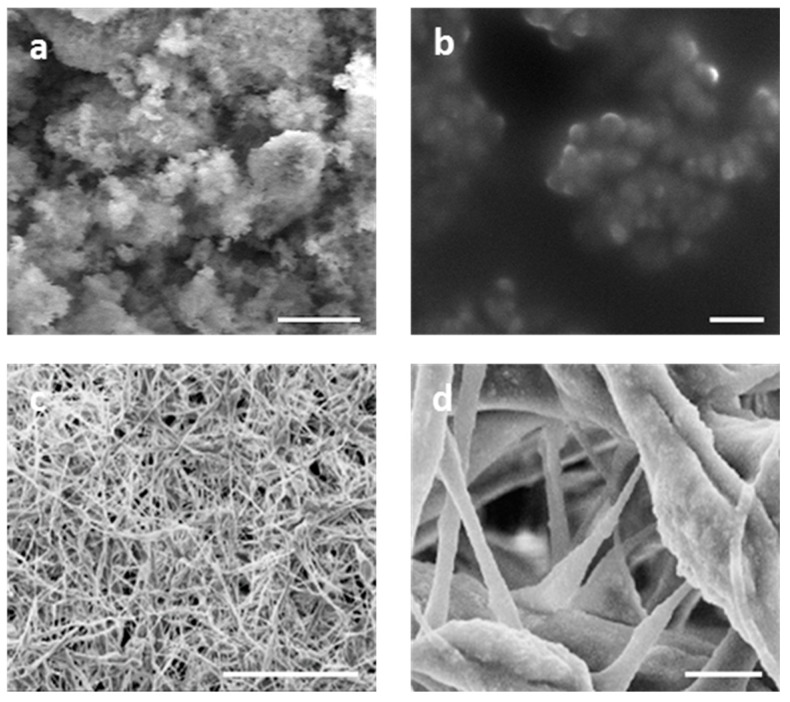
(**a**,**b**) SEM images of FeHA at different magnifications. (**c**,**d**) images of FeHA/CA at different magnifications. Scale bars: (**a**) 1 μm, (**b**) 200 nm, (**c**) 200 μm, (**d**) 10 μm.

SEM images of the FeHA/CA scaffold are reported in Figure 5, compared with the SEM and TEM images of the FeHA nanoparticles alone. Paying attention to the FeHA images, it is possible to note that the FeHA powder is composed of micrometric particles (Figure 5a) that are agglomerates of smaller nanoparticles that are clearly visible in TEM images (Figure 5b), highlighting the nanostructural nature of this magnetic powder. Noting the microstructure of FeHA/CA, several layers of micrometric string fibers comprise the scaffold. The fibers’ surfaces are rough, due to the incorporation of FeHA crystals into CA fibers, and thanks to electrospinning technique, the mat revealed a high density of tight pores that were very close to the morphology of natural cementum, a dense and avascularized tissue. The mean fiber size measured 4 ± 3 µm, with an average porosity of 44 ± 7% of the area, while the mat thickness was 200 ± 100 µm.

### 2.4. Assembling of the Graded Periodontal Scaffold

**Figure 6:**
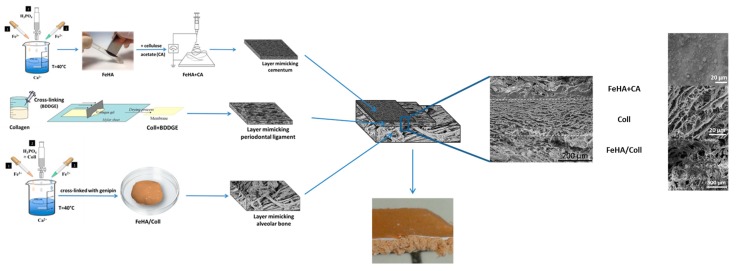
Schematic process of the FeHA/Coll–Coll–FeHA/CA tri-layer scaffold mimicking the periodontal apparatus.

The assembly of the three layers mimicking alveolar bone, PDL, and cementum into the final periodontal scaffold was obtained by the sequential deposition of precursors in the wet state, as illustrated in Figure 6. Firstly, the hybrid FeHA/Coll gel mimicking alveolar bone was spread onto a metal plate (thickness ~ 3 mm); a thin layer of Coll gel was then spread onto the first layer; finally, electrospun FeHA/CA fleece was removed from the substrate of poly(vinyl alcohol) (PVA) by soaking into deionized water, then spread on the top of the bone + ligament structure. The obtained layered construct was subjected to the freeze-drying process, giving a final periodontal-like scaffold (Figure 6). Figure 6 shows the cross-sectional morphology of the periodontal scaffold obtained by SEM analysis at various magnifications. A very compact scaffold is clearly visible with layers being well adherent between them, the bone-like layer consisting of Coll/FeHA and a ligament-like layer consisting of Coll maintained its porous structure as a cementum-like layer consisting of FeHA/CA, preserving its dense morphology.

### 2.5. Biological Characterization

**Figure 7:**
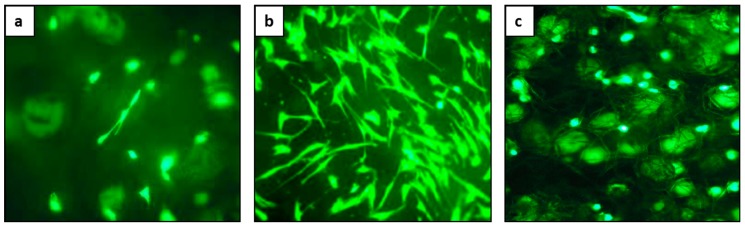
Cell viability of cell-seed FeHA/Coll (**a**), Coll (**b**), and FeHA/CA (**c**) scaffolds analyzed by the Live/Dead assay at a magnification of 20×.

In vitro evaluation was performed on each layer of the hybrid composite of Coll/FeHA, Coll, and FeHA/CA, and shows that the chemical composition and morphology of the scaffolds favor substantial cell viability and proliferation. Figure 7 shows qualitative cell viability as carried out through a Live/Dead assay after seven days. Analysis by fluorescence microscope revealed that all cells seeded on all of the three layers were alive, as reported by the intense green fluorescence related to the intracellular esterase activity of the live cells. Figure 7b shows a more widespread cell morphology for the ligament-like scaffold. Quantitative biocompatibility tests were conducted according to ISO 10993-5, and data are shown in Table 1. No cytotoxicity was highlighted in all the three materials, since cell growth inhibition is lower than 30%. FeHA/CA and Coll/FeHA exhibit values that are higher than Coll alone, and their levels were close but still lower than the minimum level allowed in the ISO standard. These tests confirm the qualitative evaluation, showing that the so-obtained periodontal scaffold can be safely applied to more complex pre-clinical evaluation tests.

## 3. Discussion

In this work, a novel approach to obtain scaffolds mimicking the multifunctional periodontal tissue complex is developed based on the basis of biological principles; in fact, the obtained scaffold was not conceived as a simple filler material, but it is designed with a biomimetic graded composition and textured structure that is able to instruct specialized cellular components of the periodontium to participate in the regenerative process. A 3D scaffold with specific properties such as tailored mineralization extent and porosity, good wettability, and stability under physiological conditions, was developed to mimic the chemical-physical properties of alveolar bone, periodontal ligament, and cementum: (i) the alveolar bone-like scaffold, featured by extensive porosity and interconnected pores to allow cell colonization and vascularization; (ii) the PDL-like scaffold, less thick and porous with longitudinally disposed fibers; and (iii) the cementum-like scaffold, obtained with close-packed and not porous structure.

The integration of different materials and techniques such as biomineralization, tape-casting, electrospinning, and freeze-drying processes allowed for an integrated scaffold to be obtained, well mimicking the composition and the morphology of the different tissues constituting the whole periodontium, with interconnected porosity connecting the layers, and all features giving a physical support for cell colonization and proliferation, and for vascularization. Biomimetic compositions and structures represent a source of chemical and morphological signals that are able to induce cell differentiation and new periodontal tissue formation.

Furthermore, our periodontal scaffold is endowed with superparamagnetic properties that are not detectable in the natural periodontium, but it is a feature that is extremely innovative and promising for advanced frontier applications in regenerative medicine. In fact, magnetic signaling from a scaffold has proven to exert an activation effect on osteoblast cells controlling bone growth and improving the whole regenerative process [27,29]. In addition, different from the common use of superparamagnetic metal oxides that elicit cytotoxicity concerns, in this work, a biocompatible, bioactive, and biodegradable magnetic phase is used. The use of biocompatible magnetic materials is promising for extending the scope of implantable devices from tissue regeneration, the delivery of magnetic carriers into the scaffold, up to imaging through magnetic resonance imaging (MRI) [27,30,31]. In addition, the presence of iron into the mineral phase gives additional advantages, positively influencing cell adhesion and proliferation, also without the presence of external magnetic fields [32]. All of these reasons justify the choice for introducing two magnetic layers into the scaffold, even if they are not present in the natural periodontium.

For the alveolar bone, collagen was selected as a biopolymeric matrix for the biomineralization process, due to its high affinity with the extracellular matrix and its well-known ability to act as a template for the heterogeneous nucleation of biomimetic inorganic phases. The final hybrid scaffold recapitulates the chemico-physical features of alveolar bone and high porosity, and exhibits superparamagnetic properties, thanks to the direct nucleation of the ion-doped apatitic phase into collagen fibrils during self-assembly, exploiting pH-dependent fibration mechanisms [15,16]. The collagen matrix imposed a strict guide of chemical features and spatial confinement, leading to the formation of a high density of nucleation sites, but a limited growth of apatitic nuclei, thus yielding nearly amorphous and nano-sized inorganic crystals. The synthesis temperature is a key aspect determining the crystallinity of hydroxyapatite and the extent of iron doping into hydroxyapatite as Tampieri et al. suggested in previous works [24,26], as it influences the kinetics of magnetite formation, apatite crystallization, and magnetization values. Therefore, *T* = 40 °C was selected as a suitable temperature to obtain a magnetic scaffold without the contribution of magnetite, and to maintain a low level of crystallinity in hydroxyapatite, conferring characteristics that are very close to natural mineralized tissues. Furthermore, the presence of a magnetic mineral phase confers interesting features to hybrid materials that under weak magnetic fields may stimulate cells to reproduce and differentiate, and in bone tissue regeneration, it highlights an osteoinductive effect, even without external magnetic forces [33,34]. Genipin, the cross-linking agent, allowed for the stabilization of the scaffold, in order to increase the resistance of the hybrid composite toward physiological fluids and bio-erosion, and thus mechanical properties [35,36]. The freeze-drying parameters were optimized to obtain the desired microstructure, which was suitable for cell colonization and proliferation.

For the PDL, the collagen scaffold was selected to produce a thin central portion of the tri-layer scaffold mimicking the periodontal apparatus. A very highly porous membrane had been developed, in order to allow an extensive vascularization of the top layer, cementum, depending metabolically by nutrients diffused from the PDL [37]. Type I collagen was the excellent candidate for its high biomimicry and biocompatibility. However, the degradation of collagen is faster than tissue regeneration, so that a cross-linking agent (BDDGE) was used to increase and modulate the stability of the scaffold in a physiological environment to an extent that is sufficient to allow cells engraftment and the deposition of new cellular matrix [38]. In the same conditions of alveolar bone, the desired structures with a high and interconnected porosity was obtained through a freeze-drying technique [13,15].

The cross-linking process is a suitable tool to increase the chemical stability of bio-polymeric and biomineralized hybrids, and their resistance to enzymatic attack, as well as to tailor fiber organization, pore size, and porosity extent. On the basis of previous studies, we selected genipin as a cross-linking agent for hybrid Coll/FeHA, and BDDGE as a cross-linking agent for the pure bio-polymeric Coll construct mimicking the PDL [13,35,39]. The ability to adjust morphological features is promising to modulate the mechanical properties and stiffness of the hybrids, and to obtain scaffolds with biomechanical features acting as instructors for the cells [40,41].

The cementum tissue is similar in structure to bone, but with a disordered and less porous structure, suitable for anchoring the tooth to its alveolus. To create a very thin layer that is morphologically and chemically close to the natural cementum, a bio-inspired process was exploited by synthesizing a non-stoichiometric nanostructured iron-doped HA (FeHA). A bio-erodible polymer like cellulose acetate was added to FeHA, and electrospun together to develop mats composed of non-woven micrometric fibers. Through an electrospinning process, a porous and very thin layer of highly mineralized fibers were obtained with favorable pore and fibers diameters for osteogenic cells [42]. In order to increase the bioactivity and bio-resorbability of the cementum layer, Fe^2+^ and Fe^3+^ ions were introduced into the HA lattice to occupy specific calcium sites, providing superparamagnetic properties [24,25]. XRD and FTIR analyses highlight that the organic phase plays a different role in the FeHA/Coll and FeHA/CA materials. In FeHA/Coll, the organic component guides mineral phase formation by heterogeneous nucleation during the biomineralization process, whereas in FeHA/CA, the organic phase is simply mixed to the mineral phase, thus obtaining an organic–inorganic composite. The constraints exerted by the collagen matrix during biomineralization induce the nucleation of the low crystalline apatite phase with very low crystallinity and a biomimetic *c*-axis orientation [15]. The mineral phase present in FeHA/CA shows a low level of crystallinity as well, but this is ascribed to the low synthesis temperature of the FeHA component, yielding a reduced growth of crystalline domains; indeed, in FeHA/CA the mineral magnetic phase was obtained by a wet process without any organic phase constraining its crystal development, as occurs in the biomineralization process.

Finally, the assembly of the tri-layered periodontal scaffold was optimized in order to preserve the desired morphology for all of the layers, and to prevent delamination. The presence of water in the still damp layers promoted their integration, creating a stable scaffold that was subjected to a unique freeze-drying process that was useful for inducing a stable level of adhesion within the single layer, and for generating a 3D functional scaffold. In vitro tests confirmed the biocompatibility, with good results obtained for each layer, and together with chemical and morphological evaluation, the tri-layer scaffold was determined to be an excellent candidate for in vivo implantation.

The periodontal region is characterized by the presence of different tissues, mineralized or not, showing composition, degree of mineralization, and morphology with very different textures. Each dental tissue is populated by different cell lineages, and they perform specific biological and biomechanical functions, in synergy with the other periodontal tissues. The achievement of biocompatible and bioactive constructs that recapitulate the relevant compositional and multi-scale structural features of native tissues is a key point to obtaining scaffolds with instructive abilities for cells. However, given the great difference between the various periodontal tissues, in the present work, we associated different manufacturing techniques capable of generating and organizing nanostructured building blocks into 3D constructs with the desired multi-scale morphology.

Biomineralization is a unique and highly versatile tool for obtaining hybrid building blocks consisting of Type I collagen mineralized with biomimetic apatitic phases, whose supramolecular assembly at the multi-scale occurs following biologic physicochemical phenomena. Cross-linking and freeze-drying processes offer flexible tools for modulating the porosity and stiffness of the final constructs, so as to obtain layers mimicking the alveolar bone and the periodontal ligament. Electrospinning is a very versatile technique that allows for the extrusion of bio-polymeric or composite materials in nano-fibrillary structures. In this work, a biocompatible and magnetically active composite was obtained in the form of a mineralized composite with disordered structure and low porosity, mimicking the characteristics of dental cementum.

The possibility of obtaining the three tissue-mimicking layers in the form of a hydrogel allowed for their interpenetration when in the wet state, and subsequent freeze drying has thus generated a scaffold with a structure reproducing, as a whole, the periodontal tissue complex. Considering that the regeneration of the periodontium is strongly dependent on the activation of a correct sequence of events involving various cell lines, which are in turn strictly dependent on the structural organization of the periodontal tissue complex, the possibility of obtaining highly biomimetic scaffolds can open up future clinical applications addressing an extremely critical and widespread clinical need, which is still largely unsolved.

## 4. Materials and Methods

Telopeptide-free type I Coll from horse tendon was supplied by Opocrin SpA, (Corlo di Formigine (MO), Italy) in the form of gel (1 wt % of Coll in acetic solution, buffered at pH = 3.5). Cellulose acetate (average Mn ~ 50,000), were purchased from Sigma-Aldrich (St. Louis, MO, USA).

Genipin (gen) with a purity of 98% was purchased from Wako Pure Chemical, Richmond, VA, USA. 1,4-Butanediol diglycidyl ether (BDDGE, 95 wt % pure) and phosphate-buffered saline (PBS) were purchased from Sigma-Aldrich. Common high-purity chemical reagents were purchased from Sigma-Aldrich (MO, USA). Ultrapure water (0.22 mS, 25 °C) was used in all experiments. 

### 4.1. Development of a Periodontal-Like Hybrid Composite

Type I collagen was selected for the synthesis of the alveolar bone and PDL, given its good physico-chemical stability, fibration ability by the pH-driven biomineralization process, and excellent biocompatibility [23]. The mineral phase, represented by Fe-doped hydroxyapatite (FeHA), was directly nucleated onto the collagen fibers during their self-assembly.

#### 4.1.1. Synthesis of the Layer Mimicking the Alveolar Bone

To avoid the oxidation of Fe^2+^ and Fe^3+^ by acetic acid of Coll, 150 g of Coll gel were fibrated with NaOH solution (0.1 M), added drop-by-drop up to pH 5.5, and extensively washed with ultrapure water by centrifugation. The assembled Coll was then re-suspended, adding 260 mL of H_3_PO_4_ (≥85 wt % pure, 0.08 M). At the same time, a basic suspension was prepared by mixing three aqueous solutions: (i) 2.71 g of Ca(OH)_2_ (≥95 wt % pure) dissolved in 500 mL; (ii) 0.689 g of FeCl_2_·4H_2_O (≥99 wt % pure) dissolved in 25 mL; and (iii) 0.574 g of FeCl_3_·6H_2_O (≥98 wt % pure) dissolved in 25 mL (total Fe = 6.2 wt %). A defined amount of iron salts was set to obtain the Fe/Ca theoretical molar ratio equal to 0.16 in the magnetic CaP phase. An acid collagen suspension was dropped into a basic suspension to synthesize the hybrid composite with a FeHA/Coll nominal ratio of 70/30 wt %, mimicking bone composition [23]. A dropwise addition procedure was carried out at 40 °C under stirring, assuring a slow pH decrease. The reaction product was kept in suspension by continuous stirring and heating for 2 h after the neutralization reaction. After the mineralization process, materials were filtered by the use of a metallic sieve (50 μm) and washed three times using ultrapure water. The material was cross-linked for 48 h at 25 °C adding a 1% (*w*/*w*) genipin aqueous solution, setting up a gen/coll ratio that was equal to 2 wt %, and washed with deionized water from the residual unreacted cross-linker. The material was finally poured into 24-well cell-culture multiwell plates and freeze-dried, setting a precise cycle: a precise cooling temperature (−40 °C) and a heating ramp (2 °C min^−1^ up to −5 and 1 °C min^−1^ up to 25 °C) at *p* = 0.1 mbar, to achieve dried porous scaffolds.

#### 4.1.2. Synthesis of the Layer Mimicking the Periodontal Ligament

The non-mineralized section of the tri-layered scaffold was produced, firstly increasing the pH of the initial acid suspension of Coll, dropping NaOH 0.5 M up to 5.5 corresponding to Coll isoelectric point where fiber assembly was at maximum. The Coll fibers was collected with the aid of a sieve, washed three times with ultrapure water, and then treated with BDDGE as a cross-linking agent through immersion for 24 h in a cross-linker aqueous solution of 1% (*w*/*w*), setting up a BDDGE/Collagen ratio that was equal to 2 wt % [38]. The resulting material was washed out from the unreacted cross-linker residues, poured into 24-well cell-culture multiwell plates, and freeze-dried.

#### 4.1.3. Synthesis of the Layer Mimicking the Cementum

Firstly, the magnetic inorganic component was synthetized (FeHA). Iron(III) chloride hexahydrate solution (17.86 g of FeCl_3_·6H_2_O in 75 mL of H_2_O) and iron(II) chloride tetrahydrate solution (12.74 g of FeCl_2_·4H_2_O in 75 mL of H_2_O), as sources of Fe^3+^ and Fe^2+^ ions respectively, were added into a suspension of calcium hydroxide (50 g of Ca(OH)_2_ in 400 mL of H_2_O) at 40 °C. A phosphoric acid solution (44.40 g of H_3_PO_4_ in 300 mL of H_2_O) was then added dropwise into the basic suspension over a period of 2 h, under constant heating and stirring, thanks to a heating mantle and mechanical stirring. The precipitate was left to ripen in the mother liquor for 24 h, and then the supernatant was eliminated and the product was washed three times with ultrapure water and centrifuged. The product was stored at 4 °C into a solution of 100 mg/L to avoid particle aggregation, and if necessary, freeze-dried and sieved through a 150 μm sieve. The total amount of iron ions with respect to calcium ions were adjusted so as to obtain Fe/Ca = 20 mol %. Regarding the production of the final FeHA/CA fleeces, first the dry FeHA was added to the solvent (composed of 80:20 wt. butanone/phenylmethanol) under constant magnetic stirring, followed by sonication for 30 min and further shaking with a vortex mixer. After this dispersion step, cellulose acetate (CA) was added in order to obtain a 26% (*w*/*w*) solution of CA with respect to the solvent, and a CA/FeHA ratio of 1:1; the mixture was shaken again until the CA dissolved completely. According to Nicosia et al. the fleeces were spun four consecutive times on a water-soluble PVA-foil (SOLVY, Gunold; thickness of 20 μm) with the following spinning parameters: needle diameter of 21 G, a spinning distance of 15.0 cm, a voltage of 5.1 kV, a feed rate of 12 mL/h, a temperature of 20.9 °C, and a relative humidity of 30% [36]. Subsequently, the fleeces were treated at 70 °C and 200 mbar in a desiccator to remove the residual solvents, especially phenylmethanol, which cannot evaporate completely during the spinning process because of its high boiling point.

#### 4.1.4. Development of the Final Periodontal Scaffold

The three layers were piled up, then a knitting procedure was applied at each interface (alveolar bone–PDL and PDL–cementum) to assure good integration by the exchange of anchor fibers between the layers, and to avoid delamination at the interface. Finally, freeze-drying with a controlled freezing temperature (−40 °C) and a heating ramp (2 °C min^−1^ up to −5 and 1 °C min^−1^ up to 25 °C) at *p* = 0.1 mbar was performed to achieve dried porous scaffolds integrating the three layers.

### 4.2. Composite Characterization

TEM observations were performed using a Tecnai F20 (FEI, Hillsboro, OR, USA) equipped with a Schottky emitter and operating at 120 and 200 keV. The powder samples, grounded in a mortar and sieved at 150 µm, were dispersed in water by the means of an ultrasonic bath, and then a few droplets of the resulting suspension were deposited on holey-carbon films supported on a conventional copper grid (300 mesh).

Additional morphological characterizations of the samples were carried out by field emission gun scanning electron microscopy (FEG-SEM) using a Sigma NTS GmbH (CarlZeiss, Oberkochen, Germany) and by environmental scanning electron microscopy (ESEM, Quanta 600 FEG, FEI Company, Hillsboro, OR, USA). The specimens were mounted on aluminum stubs using carbon tape, and they were covered with a coating of Au (Polaron Sputter Coater E5100, Watford, Hertfordshire, UK).

The X-ray diffraction (XRD) patterns of the samples were recorded by using a D8 Advance diffractometer (Bruker, Karlsruhe, Germany) equipped with a Lynx-eye position-sensitive detector (Cu Kα radiation, α = 1.54178 Å) generated at 40 kV and 40 mA. XRD spectra were recorded in a 2θ range from 20 to 80° with a step size (2θ) of 0.02° and a counting time of 0.5 s.

The magnetization curves were recorded by a Superconducting Quantum Interference Device (SQUID) magnetometer Quantum Design (San Diego, CA, USA), operating at a temperature of 8−350 K with a maximum applied magnetic field (H) of 7T. Approximately 20 mg of material were measured in a magnetic field cycle from −2T to +7T at a temperature of 310 K (close to the physiological temperature). Another magnetic test was performed by vibrating sample magnetometry (VSM) using a magnetometer MICROMAG3900, Princeton Measurements Corp. (Westerville, OH, USA) on about 10 mg of material. 

For FeHA/Coll, Fourier transform infrared (FTIR) spectra of KBr disks were collected by using a Nicolet 380 spectrometer (Thermo Fisher Scientific Inc., Waltham, MA, USA) working in the range of wavenumbers 4000–400 cm^−1^ at a resolution of 4 cm^−1^. FeHA/CA, FeHA, and CA were tested by FTIR in attenuated total reflectance (ATR) mode (Bruker Alpha, Billerica, MA, USA). A single measurement result was compiled by a 50-fold measurement between 300 cm^−1^ up to 4000 cm^−1^, using a step size of 2 cm^−1^ and an automatic background correction.

A finely ground, approximately 1 wt % mixture of the sample in KBr was pressed into a transparent disk using a hydraulic press, applying a pressure of 48.6 psi (670 MPa). A pure KBr disk was used as a blank. Calcium, phosphate, and iron contents were determined by Liberty 200, Varian (Clayton South, Australia) and, prior to the analysis, the amount of samples required to reach 20 mg of inorganic component was dissolved in 2 mL of HNO_3_, and the solution volume was increased up to 100 mL with deionized water. Reference solutions were prepared by mixing standard solutions containing the investigated atoms, and an equally diluted solution of nitric acid was also analyzed, and the corresponding spectrum was subtracted by the experimental one. The thermal properties of the FeHA/Coll sample were measured using a STA 449/C Jupiter (Netzsch, Germany). Simultaneous thermal-gravimetric analysis (TGA) and differential scanning calorimetry (DSC) were carried out on approximately 10 mg of sample placed in alumina crucibles, and then heated to 1200 °C at a heating rate of 10 °C/min under a nitrogen flow of 1 mL min^−1^. The thermal degradation behavior of FeHA/CA and FeHA were determined with a TGA-50H (Shimadzu, Kyoto, Japan). The samples were tested with rate of 5 K/min up to 600 °C in air atmosphere).

### 4.3. Cell Viability and Cytotoxicity Evaluation

The cells used for the cytotoxicity tests are mouse cells Balb/c 3T3 clone A31 (ATCC, CCL 163) or mouse mesenchymal stem cells (mMSCs; Invitrogen, Waltham, MA, USA). In order to make an initial assessment of cell viability, a qualitative test was used, the Live/Dead viability assay; this was a two-color fluorescence test that permits the simultaneous determination of live and dead cells. Cylindrical scaffolds 8.00 mm diameter and 4.00 mm high, were sterilized with ethanol and by UV irradiation. One sample was placed per well in a 24-well plate and pre-soaked in culture medium. Each scaffold was seeded by carefully dropping 20 μL of cell suspension (5.0 × 10^4^ cells) onto the upper scaffold surface, and allowing cell attachment for 30 min, before addition into each well of cell culture.

At a set time, two probes must be added to the cell culture, the calcein acetoxymethyl-esther (CAM) and the ethidium homodimer (EtD) (Molecular Probes; Live&Dead^®^ Viability/Cytotoxicity Kit, Thermo Fisher Scientific Inc.). After the incubation, the CAM was converted to the fluorescent form (intense green, λ_em_ ≈ 520 nm) by esterase that was present in metabolically active, and therefore live cells; conversely, the EtD can only permeate the damaged plasma membranes of dead cells and binding nucleic acids, making them fluorescent (bright red, λ_em_ ≈ 635 nm). Images were acquired by an inverted Ti-E fluorescence microscope (Eclipse, Nikon, Tokyo, Japan), using FITC filters (~520 nm) for CAM and TRITC filters (~650 nm) for the EtD. Qualitative analysis was performed on images with the same fire and overlapping field (FITC/TRITC), at 10× magnification after seven days of culture.

The first step in biocompatibility evaluation is the study of materials cytotoxicity which was assessed according to the ISO 10993-5 standard.

Cells were seeded in multiwell plates (six wells, 10 cm^2^, Nunc, Thermo Fisher Scientific Inc.) at a starting density of 35 × 10^3^ cells/cm^2^ in a culture medium; cultures were incubated for 24 h. Cylindrical scaffolds, 18 mm diameter and 5 mm high, were sterilized by γ-ray irradiation, and then the test material was carefully placed into direct contact with the cells in the center of each replicate wells. Wells were then incubated over a 24 h period and subsequently for another 2 min with Trypan blue (Sigma Aldrich), a blue dye that can penetrate only into the cytoplasm of dead cells; thereafter, dead cells (blue) and living cells (uncolored) were counted using a hemacytometer.

All cell-handling procedures were performed in a sterile laminar flow hood. All cell-culture incubation steps were performed into an incubator kept at 37 °C with 5% CO_2_ and at controlled humidity. Three replicates of the test material were carried out.

### 4.4. Statistical Evaluation

Results were expressed as mean ± standard error of the mean plotted on a graph. Analysis was made by a two-way Analysis of Variance (ANOVA), followed by the Bonferroni’s posthoc test. Statistical analysis was performed by Graph-Pad Prism software (version 6.0).

## 5. Conclusions

Hybrid superparamagnetic 3-layer scaffolds recapitulating the physico-chemical, morphological and structural features of periodontal tissue complex are produced by integrating different fabrication approaches. The selected techniques allow for the control of the assembly and 3D organization of nanofibrillary hybrid building blocks, composed of a bio-polymeric matrix and a biomimetic inorganic phase presenting superparamagnetic properties that enable control by a remote magnetic signal. The final scaffold reproduces the relevant features of the whole periodontium, and it is thus is a potential source of biochemical and topographical signals for the various cell lineages present in the dental environment, as instructing triggers for periodontal tissue regeneration. Besides the potential of the new scaffold to provide a tissue-conductive system that mimics the 3D environment of the periodontium, its magnetic properties, can be useful for boosting the osteogenic and osteoconductive stimuli necessary for faster and effective tissue regeneration. This functionality can be suitable, particularly in patients that are affected by reduced endogenous potential.

Therefore, the novel scaffold proposed here is promising for new regenerative therapies contrasting dental tissue impairment, provoked by various degenerative diseases such as periodontitis. In vitro investigations confirmed the excellent cytocompatibility of the scaffold and cell viability, thus encouraging further investigation towards more relevant biological studies and in vivo experiments.

## Figures and Tables

**Table 1:** Biological evaluation of the cytotoxicity of the material composing the tri-layer scaffold.

Materials	% of Viability	Results
FeHA/Coll	71	Non cytotoxic
Coll	88	Non cytotoxic
FeHA/CA	73	Non cytotoxic
